# Chidamide and decitabine can synergistically induce apoptosis of Hodgkin lymphoma cells by up-regulating the expression of PU.1 and KLF4

**DOI:** 10.18632/oncotarget.20659

**Published:** 2017-09-06

**Authors:** Tao Jiang, Fujue Wang, Lianjie Hu, Xiaomin Cheng, Yuhuan Zheng, Ting Liu, Yongqian Jia

**Affiliations:** ^1^ Department of Hematology, West China Hospital of Sichuan University, Chengdu 610041, Sichuan, China; ^2^ Department of Hematology, Sichuan Academy of Medical Sciences & Sichuan Provincial People's Hospital, Chengdu 610072, Sichuan, China; ^3^ Department of Hematology, Chengdu Military General Hospital, Chengdu 610083, Sichuan, China; ^4^ Laboratory of Hematology, West China Hospital of Sichuan University, Tianfu Life Science Park, Chengdu 610041, Sichuan, China

**Keywords:** Hodgkin lymphoma, chidamide, decitabine, epigenetic regulation, synergistic

## Abstract

Epigenetic abnormalities play important roles in the pathogenesis of Hodgkin lymphoma (HL). Highly expressed class I histone acetyltransferase (HDAC) and hyper-methylation of the promoter region of tumor suppressor genes have been demonstrated in Hodgkin lymphoma. In this paper, we investigated the synergistic effects of combination treatment of chidamide, a selective HDAC inhibitor, and decitabine, a demethylation agent, for HL cell lines and explored a new strategy for treating HL. The apoptosis rates, cell cycle, mitochondrial transmembrane potentials, epigenetic changes and gene expression of HL cell lines treated with chidamide as a single agent and in combination with decitabine were tested. We found that chidamide inhibited the proliferation of HL cells through increased apoptosis. Interestingly, after combined with decitabine, the inhibition rate and apoptotic death in HL cells were significantly increased. Further studies demonstrated that when combined with decitabine the expression of acetylated histone H3 and H3K9 induced by chidamide in HL cells increased, and also the expression of tumor suppressor genes PU.1 and KLF4, which exert inhibition through apoptosis pathway. Therefore, we could come to a conclusion that chidamide and decitabine can synergistically induce apoptosis of Hodgkin lymphoma cells by up-regulating the expression of PU.1 and KLF4. These results provide a new sight in combining two different epigenetic regulatory agents for treating HL.

## INTRODUCTION

Hodgkin lymphoma (HL) is a well-established malignancy and accounts for about 8~13% of all lymphomas [1, 2] with the classical type being the commonest. At present, chemotherapy combined with involved field radiotherapy is the standard treatment of HL and the long-term survival rate of early stage patients reaches more than 90% [3, 4]. Despite the great success there are still treatment failures and relapses to current regimen [5]. High dose chemotherapy with autologous hematopoietic stem cell transplantation showed some efficacy in the treatment of relapsed and refractory HL, but outcome is still poor for patients who were older than 45 years, did not reach complete response before transplantation or with remission period less than 1 year [6]. New drug regimens are needed for those difficult cases.

In recent years, epigenetic abnormalities caught attention in the research field of pathogenesis of HL [7–10]. Excessive activation of histone deacetylase (HDAC) and hyper-methylation of promoter region of tumor suppressor gene are two most studied aspects of epigenetic abnormalities in cancer. Class I HDAC was found highly expressed in both tumor cells and lymphocytes of microenvironment of HL and in HL cell lines [10, 11]. Promoter hyper-methylation of tumor suppressor gene is another important epigenetic abnormality of HL and two important tumor suppressor genes of HL (PU.1 and KLF4) have been found to be down-regulated through methylation changes in the promoter region in cell lines and tumor cells of HL, besides, the down regulation of KLF4 gene is related with characteristic phenotype of HL [8, 9]. HDAC inhibitors could inhibit the proliferation of HL cell lines *in vitro* through activation of caspase 9 pathway dependent apoptosis and through inhibiting and blocking Akt and STAT6 signal pathway [12]. Chidamide, the selective class I HDAC inhibitor, has been approved for the treatment of peripheral T-cell lymphoma in China, but its activity on HL has not been tested yet. Decitabine, a demethylating agent widely used in myelodysplasia syndrome and acute myelogenous leukemia [13], has been evaluated in clinical trials of lymphoid malignancies in recent years [14, 15]. *in vitro* studies showed that Decitabine could inhibit the function of MYC gene by up-regulating the expression of ID2 [16] and synergistically suppressing the proliferation of diffuse large B cell lymphoma cell lines with HDAC inhibitor Panobinostat [17]. The purpose of this study is to investigate the anti-tumor activity of chidamide and synergistic inhibition effects when combined with decitabine in HL cell lines.

## RESULTS

### Chidamide inhibits the proliferation of Hodgkin lymphoma cell lines through apoptosis pathway

**Figure 1:**
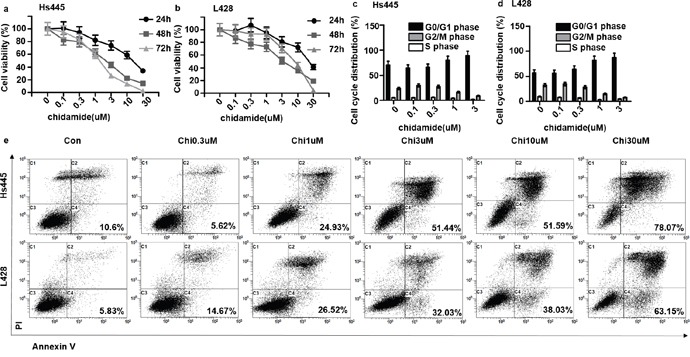
Chidamide inhibit proliferation and induce apoptosis of Hs445 and L428 cells **(a)** Proliferation rates at 24, 48, 72 hour of Hs445 cells after treated with 0.1μM, 0.3μM, 1μM, 3μM, 10μM, 30μM Chidamides; **(b)** proliferation rates at 24, 48, 72 hours of L428 cells after treated with 0.1μM, 0.3μM, 1μM, 3μM, 10μM, 30μM Chidamide; **(c)** cell cycle of Hs445 after 48 hours treatment with 0.1μM, 0.3μM, 1μM, 3μM Chidamide; **(d)** cell cycle of L428 after 48 hours treatment with 0.1μM, 0.3μM, 1μM, 3μM Chidamide; **(e)** apoptosis rates of Hs445 and L428 after 48 hours treatment with 0.1μM, 0.3μM, 1μM, 3μM Chidamide.

To observe the anti-proliferation effect of chidamide on Hs445 and L428 cell lines, 2×10^5^/mL cells were seeded in 96-well plates and treated with different concentrations of chidamide. After incubated for 24, 48, 72 hours the MTT assays were used to detect the cell proliferation rate. Results showed that after 24 hours 1μM Chidamide could inhibit both cell lines and the inhibition effect increased with increasing concentration of chidamide which reached 70% with 30μM chidamide (Figure 1a, 1b). The same concentration dependent inhibition effects of chidamide on both cell lines were found after 48 and 72hours treatment (Figure 1a, 1b). The IC50 values of Chidamide on Hs445 cells were 13.33μM at 24 hours (95%CI: 10.17-17.47), 1.91μM at 48 hours (95%CI: 1.37-2.66), 1.326μM at 72 hours (95%CI: 0.89-1.98), on L428 cell were 22.07μM at 24 hours (95%CI: 16.38-29.75), 3.46μM at 48 hours (95%CI: 2.69-4.44), 3.68μM at 72 hours (95%CI: 2.57-5.25). Cell cycle analysis revealed that 1μM and 3μM chidamide induced both cell lines arrested at G0/G1-phase after 48h of treatment (Figure 1c, 1d). Furthermore, Annexin V/PI assay revealed that after 48 hours treatment 1μM chidamide could induce apoptosis of Hs445 cell line and the effect increased with increasing concentration of chidamide. After 48 hours treatment 0.3μM chidamide could induce apoptosis of L428 cell line and the effect increased with increasing concentration of chidamide (Figure 1e). These data suggest that as a single agent chidamide can inhibit the proliferation of Hs445 and L428 cell lines through apoptosis pathway.

### Chidamide and decitabine synergistically inhibit proliferation of Hodgkin lymphoma cell lines

**Figure 2:**
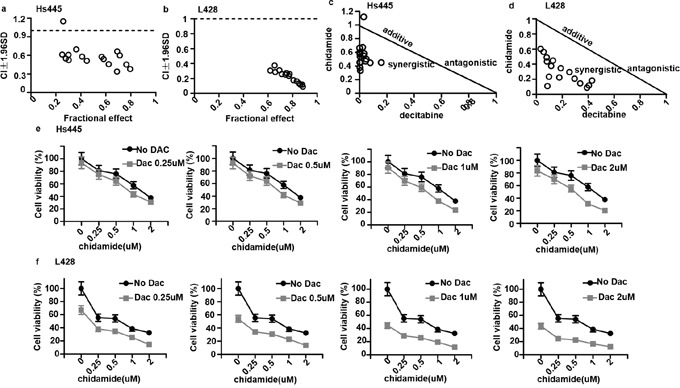
Synergy between chidamide and decitabine in Hs445 and L428 cells **(a)** CI values for combination of chidamide and decitabine in Hs445 cell line after 72 hours of incubation; **(b)** CI values for combination of chidamide and decitabine in L428 cell line after 72 hours of incubation; **(c)** CI shown in normalized isobolograme for combination of chidamide and decitabine in Hs445 cell line after 72 hours of incubation; **(d)** CI shown in normalized isobolograme for combination of chidamide and decitabine in L428 cell line after 72 hours of incubation; **(e)** Combination of chidamide and decitabine in Hs445 cell line after 72 hours of incubation. Values represent means expressed as percentages compared with the untreated control, error bars represent SD; **(f)** Combination of chidamide and decitabine in L428 after 72 hours of incubation. Values represent means expressed as percentages compared with the untreated control, error bars represent SD.

To examine whether the combination of chidamide and decitabine could induce synergistic killing of Hodgkin lymphoma cell lines *in vitro*, Hs445 and L428 cells were treated with various combinations of chidamide(0.25, 0.5, 1, 2μM) and decitabine (0.25, 0.5, 1, 2μM) for 72h. The inhibition effects were tested by MTT assays and the combination indexes (CI) were calculated by CalcuSyn software. The combination of 0.5μM chidamide and 0.25μM decitabine in Hs445 cell showed no synergism (CI=1.15>1) (Figure 2a), but other combination groups in both cell lines showed strong synergetic anti-proliferative effects (CI<1) (Figure 2b). Similarly, isobologram analysis also demonstrated this synergy of the combination in both cell lines (Figure 2c, 2d). These data suggest that combination of chidamide and decitabine synergistically inhibits proliferation in Hodgkin lymphoma cell lines *in vitro*. For the following experiments the drug concentrations were 0.5μM chidamide and 2μM decitabine for Hs445 and 0.5μM decitabine and 2μM chidamide for L428.

### Chidamide and decitabine synergistically increase apoptotic death in Hodgkin lymphoma cell lines

**Figure 3:**
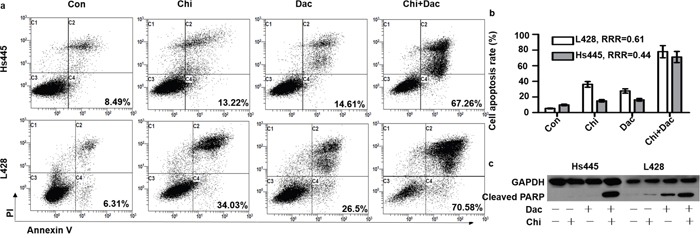
Effects of combined treatment of chidamide and decitabine on apoptosis of Hs445 and L428 cells **(a)** Apoptosis rates of Hs445 and L428 after 48 hours treatment with combination of chidamide and decitabine; **(b)** RRRs for Hs445 and L428 after 48 hours treatment with combination of chidamide and decitabine; **(c)** cleaved PARP1 expression of Hs445 and L428 after 48 hours treatment with combination of chidamide and decitabine.

**Figure 4:**
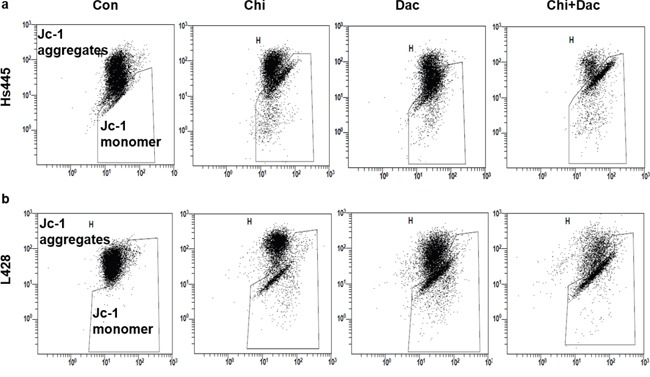
Effects of combined treatment of chidamide and decitabine on mitochondrial damage of Hs445 and L428 cells **(a)** JC-1 assay of Hs445 cells after 72 h treatment with chidamide and decitabine; **(b)** JC-1 assay of L428 cells after 72 h treatment with chidamide and decitabine.

We then investigate the synergistic effects of chidamide and decitabine on apoptosis of both cell lines. Both cell lines were seeded at 2×10^5^/mL in 12-well plates or 5cm culture dishes and treated with chidamide and decitabine alone or combined at concentrations mentioned above. Combined treatment of chidamide and decitabine resulted in higher percentage of apoptotic cells (67.26%±7.87% for Hs445 and 70.58%±5.76% for L428), when compared with either drug alone in both cell lines (p<0.05) (Figure 3a). The RRRs of two drugs for Hs445 was 0.44 and for L428 was 0.61 (Figure 3b). We also detected the expression of cleaved PRAP1. There were nearly no expression of cleaved PRAP1 in Hs445 cells treated with chidamide or decitabine alone, while the combined treatment induced its expression. Exposure of L428 cells to either drug alone could induce slight expression of cleaved PRAP1 protein, while the combined treatment strongly increased its expression (Figure 3c). Moreover, we performed the JC-1 assay to investigate whether chidamide/decitabine could affect the mitochondrial membrane potentials in both cell lines. Our data showed that the combination could effectively reduce the mitochondrial membrane potentials when compared with either drug alone or control group (Figure 4). Collectively, we demonstrated that combination of chidamide and decitabine synergistically increased apoptosis of both cell lines.

### Decitabine enhances *in vitro* activity of chidamide in Hodgkin lymphoma cell lines

**Figure 5:**
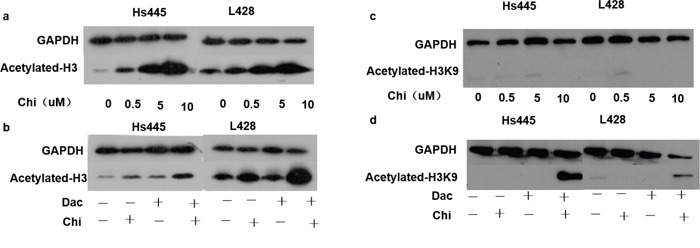
Expression of acetylated histones after treated with chidamide and decitabine **(a)** Expression of acetylated-H3 in Hs445 and L428 cells after 48 hours treatment of 0, 0.5, 5 and 10μM chidamide; **(b)** expression of acetylated-H3 in Hs445 and L428 cells after 48 hours treatment of chidamide and decitabine alone or in combination; **(c)** expression of acetylated-H3K9 in Hs445 and L428 cells after 48 hours treatment of 0, 0.5, 5 and 10μM chidamide; **(d)** expression of acetylated-H3K9 in Hs445 and L428 cells after 48 hours treatment of chidamide and decitabine alone or in combination.

To explore whether decitabine would affect HDAC activity of chidamide, 2×10^5^/mL cells were seeded in 6-well plates and treated with chidamide and decitabine alone or in combination as detailed above. After 48 hours treatment cells were harvested and proteins were extracted for western-blot analysis. Results showed that expression of acetylated total H3 in Hs445 and L428 cells increased after treated with chidamide alone in a dose-dependent manner (Figure 5a). However acetylated H3K9 was not observed in Hs445 or L428 cells treated with chidamide alone even when the concentration increased to 10μM (Figure 5b). The expression of acetylated total H3 in Hs445 and L428 cells increased significantly after treated with combination of chidamide and decitabine compared with chidamide alone (Figure 5c). Moreover acetylated H3K9 was observed in Hs445 or L428 cells treated with combination of chidamide and decitabine (Figure 5d). Taken together, our results suggest decitabine can enhance HDAC activity of chidamide in Hodgkin lymphoma cell lines.

### Combination of chidamide and decitabine changes expression of tumor suppressor genes

**Figure 6:**
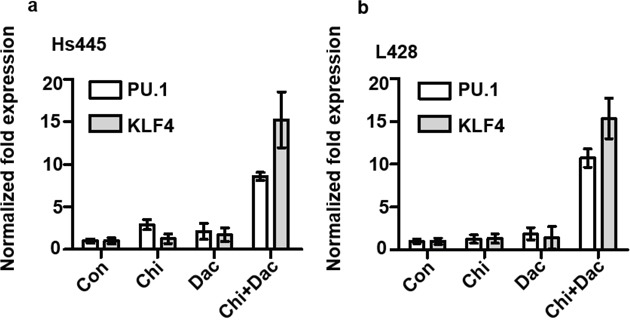
Expression of PU.1 and KLF4 gene after treated with chidamide and decitabine **(a)** Expression PU.1 and KLF4 gene in Hs445 after 48 hours treatment of chidamide and decitabine alone and combination, the concentrations were detailed in text; **(b)** expression PU.1 and KLF4 gene in L428 after 48 hours treatment of chidamide and decitabine alone and combination at indicated concentration.

PU.1 and KLF4 are potent tumor suppressor genes in HL cells. To investigate whether the expression of these two genes could be induced by the combined treatment of chidamide and decitabine, 2×10^5^/mL cells were seeded in 6-well plates and treated with chidamide and decitabine alone or in combination as detailed above. After 48 hours treatment cells were harvested, total RNAs were extracted and converted to cDNAs for Q-PCR assay. Compared to control group, the relative expression levels of PU.1 and KLF4 of Hs445 and L428 cells after treated with chidamide or decitabine alone did not increase. However the relative expression levels of PU.1 and KLF4 of Hs445 and L428 cells after treated with combined drugs significantly increased (Figure 6). The combination of chidamide and decitabine could up-regulate the expression of PU.1 and KLF4.

## DISCUSSION

In this study, we demonstrated chidamide inhibits the proliferation of Hodgkin lymphoma cell lines through apoptosis pathway and arresting cell cycle in G0/G1 phase. Furthermore, the synergism of chidamide and decitabine as two kinds of epigenetic regulatory drugs was also identified, and more importantly, we observed that decitabine could enhance the HDAC inhibitory activity of chidamide in Hodgkin lymphoma cell lines. Collectively, these results provided new insight into the treatment strategies for Hodgkin lymphoma. Previous studies indicated that HDAC inhibitor could kill HL cells *in vitro* [12, 18, 19] and was reported successful in clinical application [20]. Chidamide can selectively inhibit HDAC1, 2, 3 and 10 [21, 22], and IC50 of chidamide in HL cell lines is between 0.4μM to 40μM. Moreover, Gong K et, al [21] reported that chidamide could induce apoptotic death of leukemia cell lines through caspase-depending apoptotic pathways *in vitro* and in a concentration dependent manner and leukemia cell lines were arrested in G0/G1 phase after treated by chidamide through upregulated cyclin E1. Similarly, we demonstrated chidamide inhibited proliferation and increased apoptosis of Hs445 and L428 cells *in vitro* in time- and concentration-dependent manner, and HL cell lines were also arrested to G0/G1 phase. Hodgkin lymphoma is a malignancy originated from B cells but lost characteristics. Previous studies demonstrated that the genes related with cell death, toll-likes receptor pathway and myeloid differential are acetylated [23] and the genes related with lymph cell activation, apoptosis and tumor suppression are hyper methylated [7, 24–26]. These findings suggested a possibility to combine these two different epigenetic regulatory drugs to treat HL. Our results showed that chidamide and decitabine could synergistically inhibit Hs445 and L428 cell lines by increased apoptotic death. The finding of decreased mitochondrial membrane potential and increased apoptosis protein Cleaved PARP1 was also consistent with the aforementioned findings. Hyper-methylation in promoter of tumor repressor genes plays a significant role in epigenetic dysregulation of HL. Which contributes to downregulation of B cell specific transcription factors in HL cell lines or primary HL tumor cells and can be partially reversed by the demethylation drugs [27]. PU.1 and KLF4 are acknowledged potent tumor repressor genes in HL cell lines, which could exert inhibitory effect through apoptotic pathway [8, 9], but silenced due to the promoter hyper-methylation. Of important note is that demethylation alone cannot increase the expression of PU.1 gene in L428 cell line unless the addition of specific transcription factors [26]. Other findings also showed gene expression profiles induced by combination of panobinostat and decitabine varied from either drug alone. Panobinostat enhance the demethylation effect of decitabine followed by tumor suppressor genes upregulation and decitabine enhance the histone acetylation of panobinostat in return. Our present study showed that combination of chidamide and decitabine greatly increased the expression of PU.1 and KLF4 compared to either drugs alone, which was consistent with Yuki's report [8]. We speculated that the difference is due to histone acetylation. It is generally known that histone H3 plays a critical role in DNA transcription involved in epigenetic regulation. Acetylation of histone H3 occurs at lysine site including K9, 14, 18, 23 [27]. Acetylated H3K9 is relatively more important to gene expression in HL cell lines [23]. Gong K et. al [21] observed that chidamide could induce acetylation of histone H3K9, H3K18 and H4K8, and acetylated histone H3K9 also could be induced by chidamide in pancreatic carcinoma cell lines [28], while no effect on total H3. However, unlike the previous reports, cells treated with chidamide alone could not induce expression of acetylated histone H3K9, whereas acetylated histone H3 could be induced by chidamide in a concentration-dependent manner in HL cell lines. There is a close relationship between the function of the genome in eukaryote and post-translational modifications (PTMs) of histones. Eukaryote recognizes PTMs through specific protein to regulate gene expression, and methylation and PTMs of histones exert synergism on chromatin modifications, raising effector proteins, promoting macromolecular complexes to remote sites of genome, which suggests communication exists between DNA methylation and PTMs of histones. Kalacc et.al [17] observed acetylated histone H3 in diffuse large B cell lymphoma (DLBCL) cell lines increased after 24h and 48h treatment with panobinostat combining decitabine compared to either drug alone, which suggests decitabine can enhance the activity of HDAC inhibitor. Here, we performed the experiments in which combining chidamide with Decitabine greatly induced acetylation of histone H3 and H3K9 that coincide with the up-regulated cell apoptosis, apoptosis protein and tumor repressor genes. Collectively, these results suggest that decitabine can enhance the activity of chidamide and indicate that aberrant methylation of DNA may have a synergetic effect with the aberrant activation of histone deacetylase contributing to the pathogenesis and development of HL.

Histone modifications include acetylation, methylation, ubiquitination and phosphorylation, and these modifications deeply interact with each other. Combinatorial PTMs form a complex “modifying language” that is interpreted by PTM-specific histone-interacting protein modules [29]. Although decitabine is a DNA demethylating drug, it also decreases the methylation of histones in HL cell lines [30]. In summary, our study showed that chidamide and decitabine can synergistically up-regulating tumor repressor genes, subsequent cell apoptosis induction. This finding indicates that the drug combination exerts the synergism through changing the “modifying language” to affect the anti-oncogenes.

## MATERIALS AND METHODS

### Cell lines and cell culture

The human HL cell lines Hs445 (Cobioer Bioscience, Nanjing, China) and L428 (Cobioer Bioscience, Nanjing, China) were cultured in complete RPMI 1640 medium as described earlier [16]. Cells were treated with Chidamide (Chipscreen, Shenzhen, China) and Decitabine (Hansoh, Jiangsu, China) alone or in combination at various concentrations. MTT assay was used to measure inhibitory effect as previously described [31].

### Cell-cycle analysis and apoptosis assay

After incubation the cells were collected and washed with PBS. The cells were fixed overnight in 75% ice-cold ethanol. The cells were harvested and washed by PBS and then incubated with 50μg/ml PI (propidium iodide) and 100μg/ml RNase A for 1 h. The DNA content was analyzed by flow cytometry (Beckman Coulter). Annexin V-FITC/PI staining assay was used for apoptosis assay and performed according to the manufacturer's instructions (Four A Biotech, Beijing, China).

### Analysis of mitochondrial transmembrane potential

Changes of the mitochondrial transmembrane potential were measured by JC-1 staining flow cytometry. Hs445 and L428 cells were collected and incubated with the mitochondrial membrane potential-sensitive fluorescent dye JC-1 for 20 min at 37°C, and then centrifuged at 1000 r/m for 3 min and washed in PBS for 2 times followed by supernatant discarded, precipitate was resuspended with 200μL PBS and immediately analyzed with a flow cytometer.

### Western blot analysis

After different treatment the cells were collected and washed in ice cold PBS, added with Cocktail cell lysis solution (Roche Molecular Bio chemicals, Indianapolis, IN, USA) and 1% phosphatase inhibitors (Kaiji Biological Technology, Nanjing, China) and lysed on ice for 40 minutes. The cell lysates were then centrifuged at 12000 RPM for 20 min at 4°C and the supernatant collected. The protein levels of all samples were quantified by a BCA assay according to the manufacturer's instructions (Kangwei Century Biotechnology, Beijing, China). Protein samples were equalized and separated by SDS/PAGE and transferred to PVDF membrane (Millipore) and detected by chemiluminescent HRP (horseradish peroxidase) substrate (Millipore). The antibodies used were as following: anti-Cleaved-PRAP1 (Cell Signaling Technology); anti-[acetyl-histone H3 (Lys9)] (Abcam); anti-GAPDH (ZSGB Bio, Beijing, China); anti-[acetyl-histone H3] (Abcam).

### RNA extraction and real-time PCR

Total RNA from cell lines was extracted using Trizol (Invitrogen, Carlsbad, CA) and converted to cDNAs using Reverse Transcription kit (TaKaRa Biotechnology) according to the manufacturer's instructions. SYBR Green Q-PCR reagent (Bio-tol) was used to detect expression of two tumor suppressor genes PU.1 and KLF4. The primer for PU.1 was Forward ATGACGTGTGTTGAACAAGACA, Reverse CGATGGTTGATTAAAGCCAGGT, the primer for KLF4 was Forward CCCACATGAAGCGACTTCCC, Reverse CAGGTCCAGGAGATCGTTGAA. Reactions were performed using a Bio-Red Real-Time PCR system. The expression levels of GAPDH were used to normalize the relative expression levels of both genes.

### Statistical analyses

GraphPad Prism 5.0 software was used to calculate IC50 (half the maximal inhibitory concentration) for each cell line. Combinational indexes (CI) were calculated using Calcusyn Version 2.0 software. Values <1 represents synergistic effect of the two drugs, values equal to 1 indicates the mean additive effect of the drugs, and values >1 represents an antagonistic effect. Relative risk ratio (RRR) was also used as a model for establishing synergy between two drugs. RRR is based on calculating the ratio between the actual value and expected value (EV). In the case of 2 cytotoxic compounds, EV is calculated by formula: EV=NA×NB/100, where NA represents the percentage of viable cells in the sample treated with drug A and NB represents the percentage of viable cells in the sample treated with drug B. RRR values<1 represents the synergistic effect of the 2 drugs: values equal to 1 indicates the mean additive effect of the drugs; and values>1 represents an antagonistic effect.

## Abbreviations

HL: Hodgkin lymphoma
HDAC: histone acetyltransferase
CI: Combinational indexes
RRR: Relative risk ratio
EV: expected value
PTMs: post-translational modifications
DLBCL: diffuse large B cell lymphoma
