# Correction: Chidamide and decitabine can synergistically induce apoptosis of Hodgkin lymphoma cells by up-regulating the expression of PU.1 and KLF4

**DOI:** 10.18632/oncotarget.25302

**Published:** 2018-04-20

**Authors:** Tao Jiang, Fujue Wang, Lianjie Hu, Xiaomin Cheng, Yuhuan Zheng, Ting Liu, Yongqian Jia

**Affiliations:** ^1^ Department of Hematology, West China Hospital of Sichuan University, Chengdu 610041, Sichuan, China; ^2^ Department of Hematology, Sichuan Academy of Medical Sciences & Sichuan Provincial People’s Hospital, Chengdu 610072, Sichuan, China; ^3^ Department of Hematology, Chengdu Military General Hospital, Chengdu 610083, Sichuan, China; ^4^ Laboratory of Hematology, West China Hospital of Sichuan University, Tianfu Life Science Park, Chengdu 610041, Sichuan, China

**This article has been corrected:** The equal contribution note has been added to Drs. Jiang and Wang.

Tao Jiang^1,2,*^, Fujue Wang^1,4,*^, Lianjie Hu^1,4^, Xiaomin Cheng^1,3^, Yuhuan Zheng^1,4^, Ting Liu^1,4^ and Yongqian Jia^1,4^

^*^ These authors have contributed equally to this work

Original article: Oncotarget. 2017; 8:77586-77594. https://doi.org/10.18632/oncotarget.20659

